# Navigating the challenges: A case of iatrogenic cervical esophageal perforation following thyroid surgery

**DOI:** 10.1016/j.ijscr.2024.110223

**Published:** 2024-08-29

**Authors:** Andry Irawan, Catharina, Edbert Wong, Tommy Supit

**Affiliations:** a Universitas Pelita Harapan, Faculty of Medicine, Jl. Boulvard Jendral Sudirman No. 15N, Lippo Village, Tangerang 15811, Indonesia; b Siloam Hospitals Lippo Village, Jl. No. 6, Bencongan, Kelapa Dua, Tangerang 15810, Indonesia; c Universitas Diponegoro, Faculty of Medicine, Jl. Prof. Soedarto No. 13, Tembalang, Semarang 50275, Indonesia; d Department of Surgery, Division of Digestive Surgery. Universitas Diponegoro, Faculty of Medicine, Kariadi Hospital, Jl. Dr. Sutomo No. 16, Randusari, Semarang 50244, Indonesia; e Universitas Pelita Harapan, Faculty of Medicine, Jl. Boulveard Jendral Sudirman No. 15, Lippo Village, Tangerang 15810, Indonesia

**Keywords:** Esophageal perforation, Cervical, Iatrogenic, Thyroidectomy, Neck dissection, NPWT

## Abstract

**Introduction:**

Iatrogenic cervical esophageal perforation (ICEP) represents a rare and challenging surgical complication that results in significant morbidity and mortality. This is a case report of a cervical esophageal fistula that resulted from an iatrogenic perforation following thyroid surgery, treated with surgical repair followed by negative pressure wound therapy (NPWT).

**Presentation of case:**

A 43-year-old female was presented with an infected post-total thyroidectomy and bilateral radical neck dissection two weeks prior. Esophagography revealed contrast leakage from the anterolateral aspect of the esophagus at the level of C7-T1. Surgical debridement exposed large (4 × 1 cm) esophageal damage. The esophageal repair was performed using a sternocleidomastoids flap reinforced with BioGlue® followed by placement of a feeding tube through a gastrostomy. Subsequent mucus leakage was observed two weeks after the repair. Conservative approach using NPWT was used to promote wound closure. Over a 3-week period, serial imaging demonstrated fistula closure and complete wound healing by 8 weeks.

**Discussion:**

A delay in diagnosis and treatment for esophageal perforation leads to severe complications, highlighting the need for standardized treatment algorithms. Larger perforations illustrated in this case report require primary repair with muscle flaps. When reconstructive surgery fails, a follow-up conservative therapy utilizing proper NPWT was able to heal the leakage.

**Conclusion:**

This case report illustrates rare but devastating potential complications from a commonly performed oncologic surgery. The involvement of a multidisciplinary team from the very first identification of surgical complications is crucial for ensuring proper treatment.

## Highlights

• Iatrogenic cervical esophageal perforation (ICEP) is a rare but serious complication from thyroid surgery.
• Early surgical repair of ICEP is crucial to improve the success rate of treatment and patient outcome.
• Smaller ICEP defect without systemic signs may be managed conservatively; larger ones require surgical repair.
• BioGlue® fand NPWT are innovative techniques that significantly aid in the successful management of ICEP.

## 1 Introduction

Iatrogenic cervical esophageal perforation (ICEP) represents a rare and challenging surgical complication that results in significant morbidity and mortality [[1], [2], [3]]. Among the various etiologies, iatrogenic injuries contribute to 46.5 % of all the cases. The majority of ICEP was caused by endoscopic procedures, accounting for an incidence of 0.03 % to 2 % [1,2]. A rarer etiology for ICEP were surgical procedures performed near the esophagus, such as thyroidectomy or neck dissections. Within the past two decades, only a handful of case reports documented ICEP following a thyroidectomy [[1], [2], [3], [4], [5], [6], [7], [8], [9]]. The implications of such occurrences underscore the necessity for meticulous surgical technique and heightened vigilance during postoperative care. This is a case report of cervical esophageal fistula that resulted from an iatrogenic perforation following thyroid surgery, surgically repaired using local muscle flaps followed by negative pressure wound therapy (NPWT). We elaborate the importance of proper surgical management, a collaborative approach, and the usage of innovative wound treatment strategies to increase the chance of successful management. The patient was managed in an academic hospital and presented in this report based on the SCARE criteria [10].

## 2 Presentation of case

**Fig. 1:**
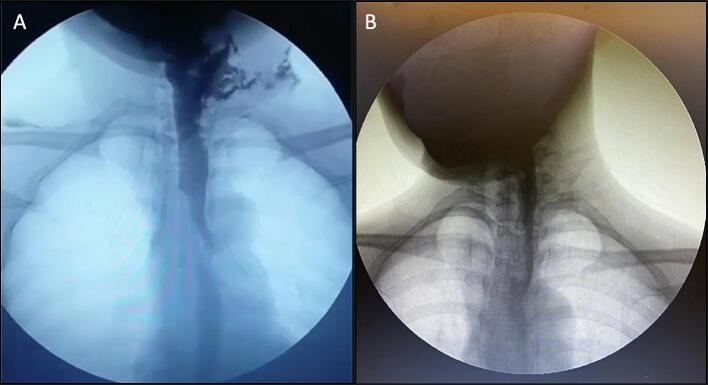
A: Esophagography showing contrast leakage from cervical esophagus, B: follow up esophagography 5 weeks post operation (after 3 weeks of NPWT).

A 43-year-old female was brought into the emergency department by family members with a chief complaint of a painful post-operative wound. Two weeks prior, the patient was diagnosed with a follicular variant of papillary thyroid carcinoma (AJCC 8th T4aN1bM0) and underwent total thyroidectomy with bilateral modified radical neck dissection type III. The patient experienced ICEP during the surgery, and the esophagus was repaired by primary suturing by the oncologic surgeon. The patient was given oral diet through a nasogastric tube (NGT) and discharged. Initially, the patient was able to tolerate liquid diet (milk); however, after one week she complained of neck pain, worsening dysphagia, and pus discharge from the wound. Fourteen days later, the patient was presented in the emergency department with painful postoperative wound on the left side with pus discharge and erythematous skin. Other symptoms include mild fever, and general malaise for the past week. Abnormal laboratory results include leukocytosis (17.800 cells/mm^3^), low hemoglobin (11.50 g/dL), and low hematocrit (34.9 %). Esophagography showed contrast leakage from the anterolateral aspect of esophagus at the level of C7-T1 with extension to the left paramediastinal space, and fistulation to the skin surface at the left supraclavicular region (Fig. 1A).

Urgent surgical debridement by a board-certified digestive surgeon exposed a large esophageal defect of 4 cm in longitudinal and 1 cm in transverse length, a partially intact mucosal layer, exposed NGT with significant inflammation of the surrounding tissue. The surgical repair was performed by debridement of necrotic tissue, primary suture of the esophageal defect, application of BioGlue® adhesive, and coverage using the left sternocleidomastoid muscle. A gastrotomy and feeding tube placement were performed to enable early enteral feeding. The patient was treated in the ICU for 2 days before being transferred to the general ward. The patient suffered from subsequent leakage and wound dehiscence from the same wound site on day fourteen, without symptoms of systemic infection.

**Fig. 2:**
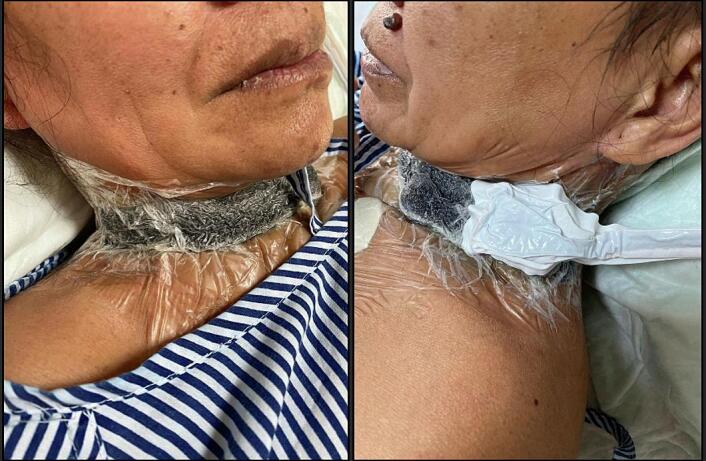
Post-operative wound covered with NPWT.

**Fig. 3:**
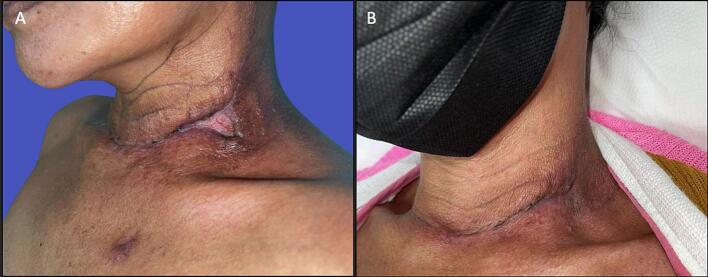
A: Clinical photograph at 5 weeks post operation, B: 8 weeks post operation.

Conservative treatment was employed using NPWT (Renasys, Smith and Nephew) at 180 mmHg with weekly dressing changes (Fig. 2). The wound pus culture showed growth of *Acinetobacter baumannii*, which was sensitive to Cefoperazone/Sulbactam. After three weeks, follow-up esophagography demonstrated complete closure of the fistula (Fig. 1B), small dehiscence at the left side of the neck with healthy granulation tissue (Fig. 3A). The NPWT system was taken off after four weeks. Complete healing was achieved after 8 weeks (Fig. 3B).

## 3 Discussion

Iatrogenic cervical esophageal perforation (ICEP) following thyroidectomy presents a significant clinical challenge due to its rarity and potential for serious complications [[1], [2], [3]]. Primary factors predisposing patients to ICEP are underlying esophageal disease and the experience of the surgeon performing the procedure [1]. Underlying esophageal disease can weaken the tissue, increasing its susceptibility to perforation during surgical manipulation. Early recognition of ICEP is crucial for initiating timely diagnostic evaluation and treatment.

The patient in this case report was presented with the classic signs and symptoms of ICEP, including neck pain, cervical tenderness, and dysphagia. Prompt identification of these clinical manifestations can expedite diagnostic imaging, such as contrast esophagography, leading to an early intervention. The choice of diagnostic modality is essential in confirming the diagnosis of ICEP. While neck radiography can detect most cases, contrast esophagography remains the gold standard due to its ability to localize and assess the size of the perforation accurately. Water-soluble contrast agents are preferred to minimize the exacerbation of inflammation and facilitate visualization of the perforation site [1]. The intraoperative injury recognition and direct repair by primary suturing in this case is justified if the perforation is less than 1 cm in size.

The management of ICEP remains controversial, with conservative and surgical approaches being employed based on the clinical scenario and institutional preferences [1,3]. Conservative management, including intensive-care unit admission, combined enteral and parenteral nutrition, antibiotic therapy, and percutaneous drainage, is often sufficient for small (3 to 7 mm), uncomplicated perforations [1,11]. Studies have shown favorable outcomes with conservative therapy, particularly in proximal or cervical esophageal perforations with limited contamination [11,12]. Larger perforations or those not expected to heal spontaneously, require surgical repair. Primary repair with wide exposure into the retropharyngeal and retroesophageal spaces are essential for successful repair [1,12,13]. Flaps using omohyoid or sternocleidomastoid muscle, can be utilized to reinforce the repair, especially for larger fistulas [1,13]. Careful consideration must be given to the cosmetics and potential functional complications associated with sternocleidomastoid muscle flap reconstruction [1,13,14]. In this case, the sternocleidomastoid muscle flap was chosen due to the large defect (4 × 1 cm) and severe inflammation, which caused adhesions in the neck structures. While this approach is ideal for managing large ICEP, as seen in this report, it presents significant technical challenges, requires careful pre-operative planning, and necessitates multidisciplinary surgical expertise.

Gastrostomy permits more physiologic feeding than jejunostomy, enabling the administration of a wider range of enteric formulas, complex nutrients, larger volume, and bolus feeding. It ensures consistent nutritional support during the postoperative recovery. The rationale for using BioGlue® to cover the defect was to provide additional structural support to prevent leaks [15]. The author considered that the protective sealing effect of the BioGlue® outweighed the potential inflammatory complications or adhesive embolization [16]. A conservative approach using NPWT was chosen to treat the recurrent fistula formation 2 weeks post-operative repair. A conservative approach was chosen because the patient does not exhibit systemic manifestations of infection. Furthermore, subsequent surgical exploration was deemed to be risky since further debridement would potentially enlarge the esophageal defect and removal of the compromised muscles. The primary effects of NPWT include wound shrinkage when the suction is applied, leading to the drawing together of wound edges and microdeformation. These changes, along with fluid removal and debridement, contribute to the alteration of the wound environment, fostering tissue approximation and removal of nonviable tissue [17,18]. Subsequently, secondary effects such as neurogenesis, angiogenesis, hemostasis, and modulation of inflammation are triggered, ultimately promoting enhanced wound healing and tissue regeneration [17,18].

Endoscopic management of iatrogenic cervical esophageal perforation (ICEP) is an evolving approach that offers potential benefits but currently lacks extensive data to support its widespread adoption as the standard of care [1,18]. One innovative endoscopic approach gaining attention is Endoscopic Vacuum Therapy (EVT), which utilizes NPWT principles to manage esophageal defects [1,[18], [19], [20]]. with reported success rates as high as 89 % [20]. The NPWT involves placing a porous material into the wound, connected to a drainage port sealed with adhesive. A controlled vacuum pump applies negative pressure ranging from −50 mmHg to −125 mmHg, leading to primary and secondary wound effects [18,20]. EVT represents a promising advancement in the management of ICEP, offering the potential for effective wound healing while minimizing the need for invasive surgical interventions.

Despite advancements in diagnosis and treatment, ICEP still carries a significant mortality rate, emphasizing the importance of early recognition and prompt intervention. Delays in diagnosis and treatment can lead to severe complications, including mediastinitis, sepsis, and multi-organ failure [1]. Collaborative approach involving oncology, digestive, thoracic surgeons, gastroenterologists, wound care specialists, nutritionists, and rehabilitation team will maximize the chance of successful treatment. Additional research and case studies are essential to standardize treatment protocols and improve clinical outcomes for patients with ICEP.

## 4 Conclusion

This article reports a rare occurrence of ICEP following a thyroidectomy and neck dissection. The initial surgical treatment using local muscle flap and fibrin sealant subsequently failed, prompting a conservative approach using NPWT system. This case report highlights the importance of early diagnosis, proper surgical management, a collaborative approach, and the usage of innovative wound treatment strategies to increase the chance of successful management.

## Consent

The patient provided written informed consent. The information in this case report has been anonymized as to prevent harm to the patient or her family. A copy of signed document of this statement is available for review on request.

Written informed consent was obtained from the patient. Information within the paper has been sufficiently anonymised not to cause harm to the patient or their family. A copy of a signed document stating this is available for review by the Editor-in-Chief of this journal on request.

## Ethical approval

Case report does not constitute research at our institution, thus ethical approval exempted by Siloam Research Ethics Committee.

## Funding

Nothing to declare.

## Author contribution

Andry Irawan: performed operation, conception of report, data collection, data analysis, manuscript writing, revision, and submission.

Catharina: conception of report, data analysis, manuscript writing, and revision.

Edbert Wong: conception of report, data analysis, manuscript writing, and revision.

Tommy Supit: conception of report, data analysis, manuscript writing, and revision.

## Guarantor

Andry Irawan.

## Conflict of interest statement

We declare no conflict of interest.
